# Secure and dynamic access control for the Internet of Things (IoT) based traffic system

**DOI:** 10.7717/peerj-cs.471

**Published:** 2021-05-10

**Authors:** Muhammad Umar Aftab, Ariyo Oluwasanmi, Abdullah Alharbi, Osama Sohaib, Xuyun Nie, Zhiguang Qin, Son Tung Ngo

**Affiliations:** 1 Network and Data Security Key Laboratory of Sichuan Province, University of Electronic Science and Technology of China, Chengdu, China; 2 Department of Computer Science, National University of Computer and Emerging Sciences, Islamabad, Chiniot-Faisalabad Campus, Chiniot, Pakistan; 3 School of Information and Software Engineering, University of Electronic Science and Technology of China, Chengdu, China; 4 Department of Information Technology, College of Computers and Information Technology, Taif University, Taif, Saudi Arabia; 5 School of Information, Systems and Modelling, University of Technology Sydney, Sydney, Australia; 6 ICT Department, FPT University, Hanoi, Vietnam

**Keywords:** Secure IoT, Dynamic access control, Attributed RBAC, Machine learning, Social computing

## Abstract

Today, the trend of the Internet of Things (IoT) is increasing through the use of smart devices, vehicular networks, and household devices with internet-based networks. Specifically, the IoT smart devices and gadgets used in government and military are crucial to operational success. Communication and data sharing between these devices have increased in several ways. Similarly, the threats of information breaches between communication channels have also surged significantly, making data security a challenging task. In this context, access control is an approach that can secure data by restricting unauthorized users. Various access control models exist that can effectively implement access control yet, and there is no single state-of-the-art model that can provide dynamicity, security, ease of administration, and rapid execution all at once. In combating this loophole, we propose a novel secure and dynamic access control (SDAC) model for the IoT networks (smart traffic control and roadside parking management). Our proposed model allows IoT devices to communicate and share information through a secure means by using wired and wireless networks (Cellular Networks or Wi-Fi). The effectiveness and efficiency of the proposed model are demonstrated using mathematical models and discussed with many example implementations.

## Introduction

**Figure 1:**
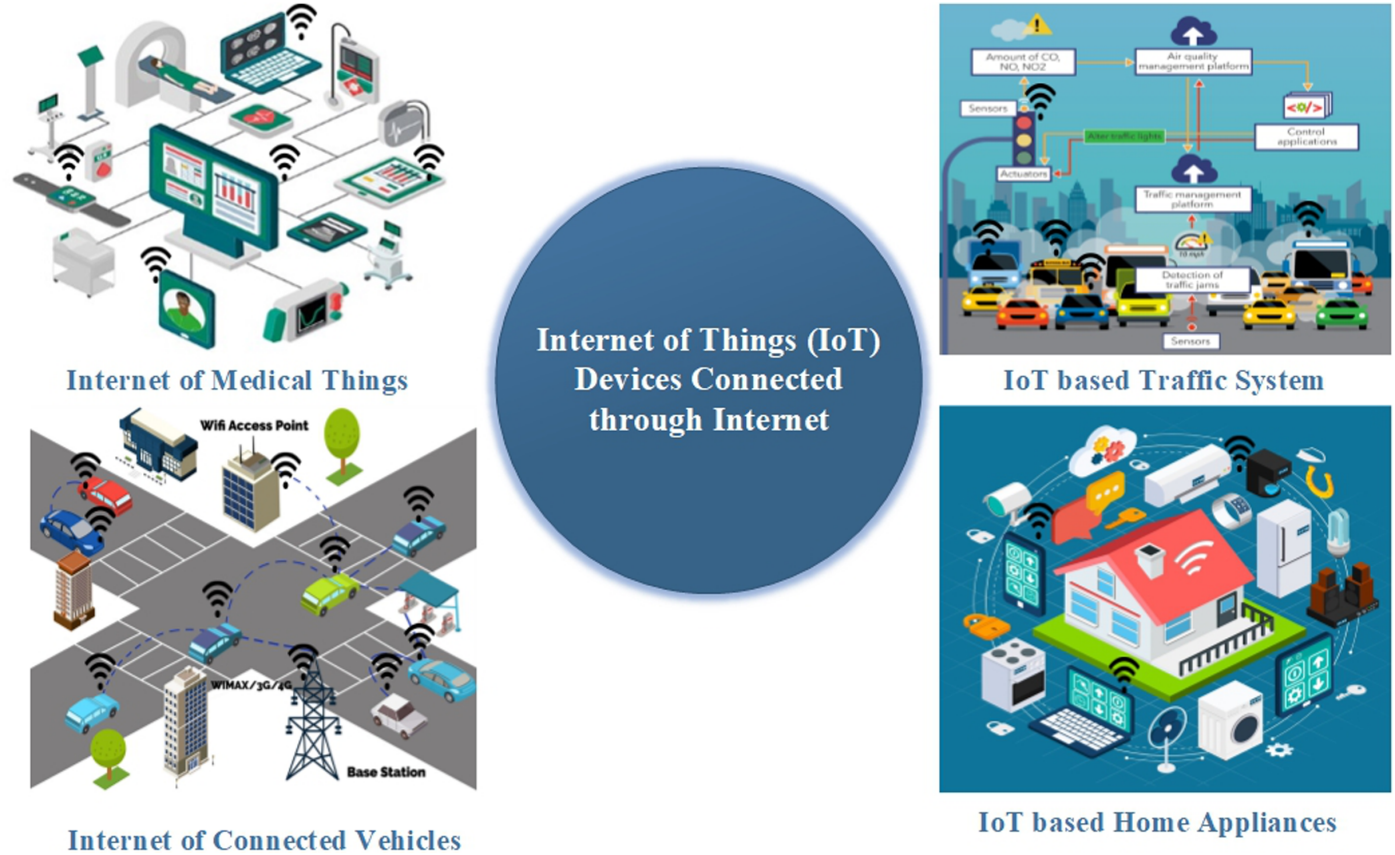
Different devices connected through internet in IoT based systems.

Internet of things (IoT) is increasingly gaining more attention due to the increase in the use of IP-based home appliances, including medical and mobile devices. Smart devices are widely used in banking, shopping, and military communication. Figure 1 shows systems based on IoT-like traffic systems, home appliances, medical equipment, and vehicle communication. Today, data security has become a challenging task for security officials, such that data security concerns have been raised during information sharing and communication (Ahanger & Aljumah, 2019). More specifically, communication between sensitive devices or vehicles is highly confidential. Therefore, the information must be secure during communication. It means some IoT devices’ security should attain high encryption according to the required security level. On the other hand, some IoT devices’ security can be implemented on a lighter note or low levels (Habib et al., 2019).

IoT trend is becoming popular day by day. The Traffic Signal Systems (TSS) is also operated through the internet so that administrators can easily manage traffic while sitting in their offices. In this regard, the TSS’s security is essential, especially when some government or military officials are passing through it. Security agencies focus on the security of vehicles and routes so that the sensitive objects (i.e., bank cash, weapons, and costly equipment) and government and military officials (i.e., Prime Minister, President, and Army Chief) are safe. They purchase bullet-proof vehicles for the officials, and the whole route is pre-defined with adequate backup routes. If an attacker or unauthorized person attacks the TSS, then the attacker can jam the entire city. This way, some criminal elements can attack the vehicles for robbery or any other ill deed as the vehicles remain stuck in congestion. Therefore, there is a need for secure and dynamic access control in traffic systems.

In this paper, a comprehensive review of various articles is conducted in the field of IoT-based systems. More specifically, the study and analysis are based on access control that lies under privacy and security. Various researchers published their work in top journals and conferences in the security of internet-connected vehicles, the internet of medical things, and the cloud’s integration with IoT. There is an absence of a dynamic and secure access control model for the traffic signals that are based on IoT. In this way, a hybrid model is proposed that is a merger of role-based access control (RBAC) (Kalinin et al., 2018) and attribute-based access control (ABAC) (Ding et al., 2019). Access control is an approach in which a structure gives or rescinds the right to access certain information or to perform a particular action. In this case, the data must be safeguarded from hidden security dangers since a massive share of coercions often arises from within a firm (Aftab et al., 2015). Access control could be referred to as security bound applied contrary to inner security coercions. The RBAC operates as a progression in the scope of access control (ANSI/INCITS, 2004). For the RBAC, the primary concept is the role that can validate the access control strategy for a particular firm or enterprise.

Permissions are generated when actions are applied to objects. Then permissions are assigned to roles and roles are assigned to users. In this case, the users are not unswervingly apportioned to permissions. Moreover, the role is a link between permissions as well as users. Also, roles are used to manage several permissions efficiently. Users are apportioned to quantified roles to obtain diverse permissions (Sandhu, 1998; Cruz, Kaji & Yanai, 2018). RBAC is eminent due to its firm safety, vigorous access control facility, besides giving simplicity of controlling for the administrator. Simultaneously, role configuring and designing an explicit firm's access control coordination is a multifaceted and hard role in RBAC (Kuhn, Coyne & Weil, 2010). In this connection, some researchers have identified RBAC limitations such as violations in the separation of duties by administrators and end-users (Li et al., 2018; Habib et al., 2014).

In ABAC, access to objects or resources by subjects could be decided by diverse attributes; for example, IP address, designation, location, and time amongst others (Jha et al., 2018). The main concern behind the ABAC is not to openly assign permissions between users and objects, in its place, it permits all the permissions in regards to attributes (Xu et al., 2018). An attribute undertakes a crucial task in ABAC for giving permissions to accredited users. For instance, the location, IP address, designation, time, and date are the attributes. The value of the attribute must determine whether a user is approved for a precise resource or not. This is because the users can similarly be referenced as subjects (Hu et al., 2014). The ABAC is a flexible concept that is moderately stress-free to manage as compared to RBAC (Hu et al., 2015). The ABAC offers the simplicity of access control configuring, vibrant, and ease of access control arrangements for a firm. Nevertheless, scrutiny of roles or substitution of the users' authorizations could be challenging or problematic in ABAC (Al-Kahtani & Sandhu, 2002; Jin, Krishnan & Sandhu, 2012). Mutually, ABAC and RBAC have certain insufficiencies. It is evident that RBAC has the simplicity of organization besides the scrutiny of access control structure; nonetheless, it similarly has intricate design matters concerning the role structuring for explicit firms. The reason is the sizeable organizational structure takes a lot of time to design roles for the users. It is also hectic for the administrator to create one by one role for the users. Correspondingly, the ABAC concept has the simplicity of designing and the role structuring; however, scrutinizing, besides running it, is a problematic responsibility. There is the absence of an access control model that gives simplicity of role structuring and secure setting up of an institution's access management system on top of scrutinizing or altering user permissions. Researchers and experts specifically suggest the integration of RBAC and ABAC (Rajpoot, Jensen & Krishnan, 2015; Umar Aftab et al., 2018; Aftab et al., 2019).

Some authors have deliberated on certain tactics concerning the merger. Still, they have not proposed a complete model concerning basic (roles and permissions assignment to users) and advanced RBAC features (separation of duties and role hierarchy). Similarly, they discussed the defects that could ensue on joining ABAC and RBAC together (Kuhn, Coyne & Weil, 2010). Within this area, the introduction of attributes in the RBAC model is the main contribution. The classification of users progressively is one of the desirable strategies. The complicated classification is focused on users and positions. In the conventional RBAC model, researchers have given user definition and functions attribute (Al-Kahtani & Sandhu, 2002).

Nevertheless, with the aid of attributes, the layout was limited to users and roles. Whereas, considering the shortcomings besides the dodges, a novel model could be established that may offer equally ABAC and RBAC benefits. RBAC is a renowned model due to its security sturdiness and the ease of managing for permissions in addition to roles. However, role structuring is a chaotic work in RBAC. In the meantime, the ABAC offers ease of placement of access control structure with attributes.

Nonetheless, it does not provide the simplicity of managing for an administrator. Thus, there can be a model that may lead to safeguard system, simplicity of management for administrators, and the ease of role structuring with dynamic nature. This work is the extension of our previously proposed work (Aftab et al., 2015). The contributions of this paper are as follows:

- This work presents the design and framework of a secure and dynamic access control model for the IoT-based TSS that provides tight security due to objects and actions attributes. In this way, the permissions can restrict the users more strictly.
- The permission creation, performance analysis concerning the number of roles’ assignment, each permission assignment time, and memory consumption of each entity are performed. Our proposed work performed significantly better as compared to previously proposed models.
- This work is implemented as a prototype of the SDAC model that is definitely increased in understanding this work in a significant way. Furthermore, the example scenarios are also provided for describing the methodology from another perspective.

The organization of this paper is as follows. The next section discusses the state-of-the-art-work present in this area of research. Also, another section addresses the SDAC model. Moving forwards a new section presents the methodology of the model. Finally, the last section presents the result along with an analytical discussion.

### State of the art

To the best of the authors’ knowledge, there is no availability of a secure and dynamic access control model for IoT-based traffic systems to secure and dynamically handling data sharing and communication. More specifically, it is necessary for secret and vital information communication and sharing to be done through some secure and dynamic system. In this context, access control can be implemented efficiently. In the related literature, the proposed models are not dynamic for IoT-based traffic systems. A secure access control model has been recently proposed, but it provides security officials’ secure communication while traveling in vehicles. The model is implemented on permissions rather than roles, for the internet of connected vehicles (Habib et al., 2019). Moreover, other security and privacy issues and violations relating to the internet of vehicles, have been addressed. Mainly, the focus of research is more on the location privacy of mobile users (Joy & Gerla, 2017).

A distributed traffic control system has been proposed in recent years that work without traffic signals. The system is implemented with the concept of the internet of agents and discussed briefly with a case study. In this system, the connected vehicles can communicate with each other so that the traffic flow becomes smoother as compared to an existing system (Bui & Jung, 2018). Also, different case studies exist related to security and privacy threats in IoT-based devices and systems (Yaqoob et al., 2017). Besides, several design-level challenges have been highlighted for vehicular communications by using the 5G building blocks (Shah et al., 2018). In this connection, a framework has been proposed by researchers to avoid the messages of malicious vehicles. That framework allows only authorized vehicles to communicate and pass messages related to traffic events (Tian et al., 2019).

Researchers have also recommended numerous concepts for improving ABAC (Aftab et al., 2019; Zhang, Zheng & Deng, 2018), such as inserting the idea of roles in the ABAC model so that the number of attributes can be managed efficiently. For the RBAC model, role is of great importance, manually assigned to operators in the archetypal RBAC concept. Mostly, researchers have recommended a concept that routinely assigned operators to roles by employing their features. For instance, operators have specific attributes like location, name, and age. All the operators would be routinely apportioned to their quantified tasks regarding their features, like operators of age 16–20 years can be allotted to their distinct task; this could be referred to as role 1. Those aged 21–25 can be assigned role 2, while the remaining operators of age more than 25 years to be assigned Role 3 (Al-Kahtani & Sandhu, 2002).

There are specific methods that combine ABAC and RBAC because RBAC is stress-free to run and offers significant scrutiny of go-ahead; however, its role structuring is problematic. Additionally, ABAC gives efficient task structuring, but the breakdown of authorizations after allotting to users is hard-hitting as opposite to RBAC. Alternatively, there is a different approach for the union of RBAC and ABAC that resolves the drawbacks of two concepts and offers a further vigorous, active, significantly better role structuring as well as the scrutiny of permission for the administrator (Kuhn, Coyne & Weil, 2010). Another fine-grained access control model has been recently introduced by merging RBAC and ABAC entities so that flexibility, fine granularity, and efficiency can be achieved (Qi, Di & Li, 2018). However, these models are not designed for IoT-based systems.

An access control model for IoT-based healthcare and medical devices also exists that prevents unauthorized access. In this system, only authorized users can access the system after verification through security access tokens (SAT). SAT is proof of an authorized user, as well as, SAT is cryptographically protected (Hossain et al., 2018). A secure and efficient access control scheme is designed for the internet of medical things–primarily based on fog/cloud computing. It is capable of providing high-level security with a short execution time, for the information stored on the cloud (Wang et al., 2018). The integration of cloud computing and IoT has many security issues and challenges that are discussed in the IoT and cloud computing survey (Stergiou et al., 2018). Furthermore, various researchers working on the security and privacy of database-as-a-service, cloud-based secure services, and blockchain, along with the legitimate user recognition, challenges, and performance analysis (Khan et al., 2020; Khan et al., 2019; Ahmad et al., 2018).

### SDAC model

Due to both the models’ weaknesses and limitations, the merger of RBAC and ABAC is favored. The SDAC model is an efficient solution in the form of a hybrid model that covers flaws significantly. This model aids in resolving the problems of RBAC (ANSI/INCITS, 2004) and ABAC (Hu et al., 2015) standards, discussed in the earlier section. An administrator usually creates several permissions for the users in an organization so that implementation of the access control is fulfilled in a significant manner. Normally, the administrator creates permissions manually and one by one. This is a time-consuming and challenging job. Under this model, authorization (permissions) are automatically generated by joining the action-level and the object-containers. Authorization is generated when action and object are joined together. The concept encompasses diverse security heights, and each one of them includes certain quantified whereabouts. For level 1, it contains approve, write, print, and delete actions. For level 2, includes writing, edit, and read actions. Then, level 3 has submitted and execute actions only.

**Figure 2:**
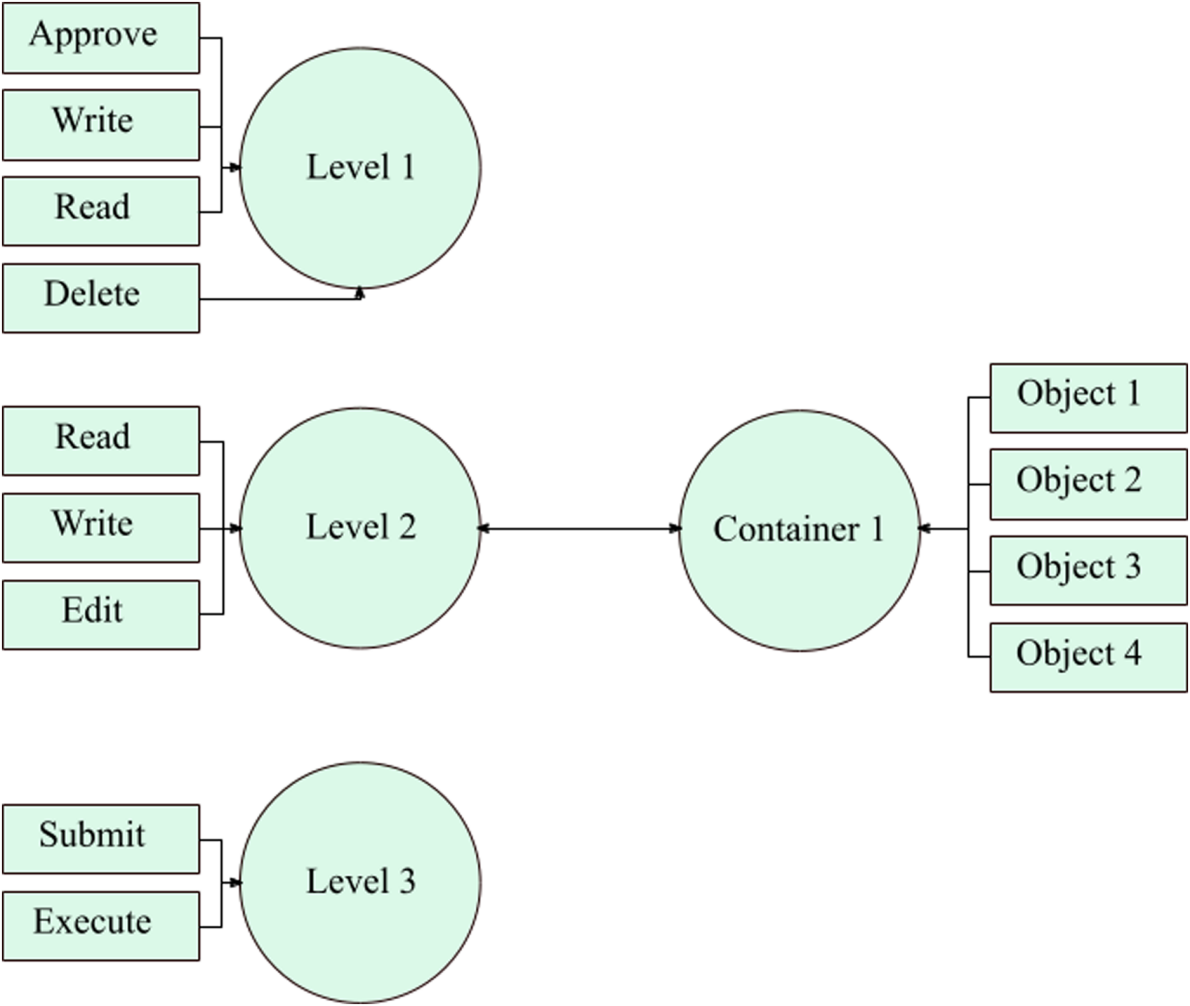
Multiple permission creation process by using object containers and action levels.

These action-levels have been prearranged along with the organizational structure. The overseer could generate additional action-levels if required. Likewise, object ampules have been shaped for the storing of objects. Several ampules could be molded for the storage of objects. Objects are usually allotted to containers concerning the category of the object. In a situation where these objects are apportioned to ampules, then the object-container would relate on an action-level. This procedure routinely produces several authorizations. According to Fig. 2, when an administrator applies action-level two on object-container 1, 12 permissions will be created at once. In this way, an administrator can create multiple permissions at a time. On the other side, the administrator has to create permissions one by one, in the traditional RBAC model. Furthermore, an IoT-based wireless sensor network can be deployed along roadside to monitor and control traffic, as discussed in (Masek et al., 2016).

The network model for sensors inter-operability can be either based on machine to machine communication (M2M) or wireless sensor network based on LoRa (low-power wide-area network technology). The IoT sensing devices can help to provide real-time traffic control along with roadside parking management solutions. There are some limitations in the deployment of these IoT sensors devices along roadside. However, there is a big challenge of providing a secure, fast, and reliable access control mechanism for the secure and reliable operation of these IoT sensors. Therefore, we propose a novel and dynamic access control model that can provide significantly better security for traffic control systems.

**Figure 3:**
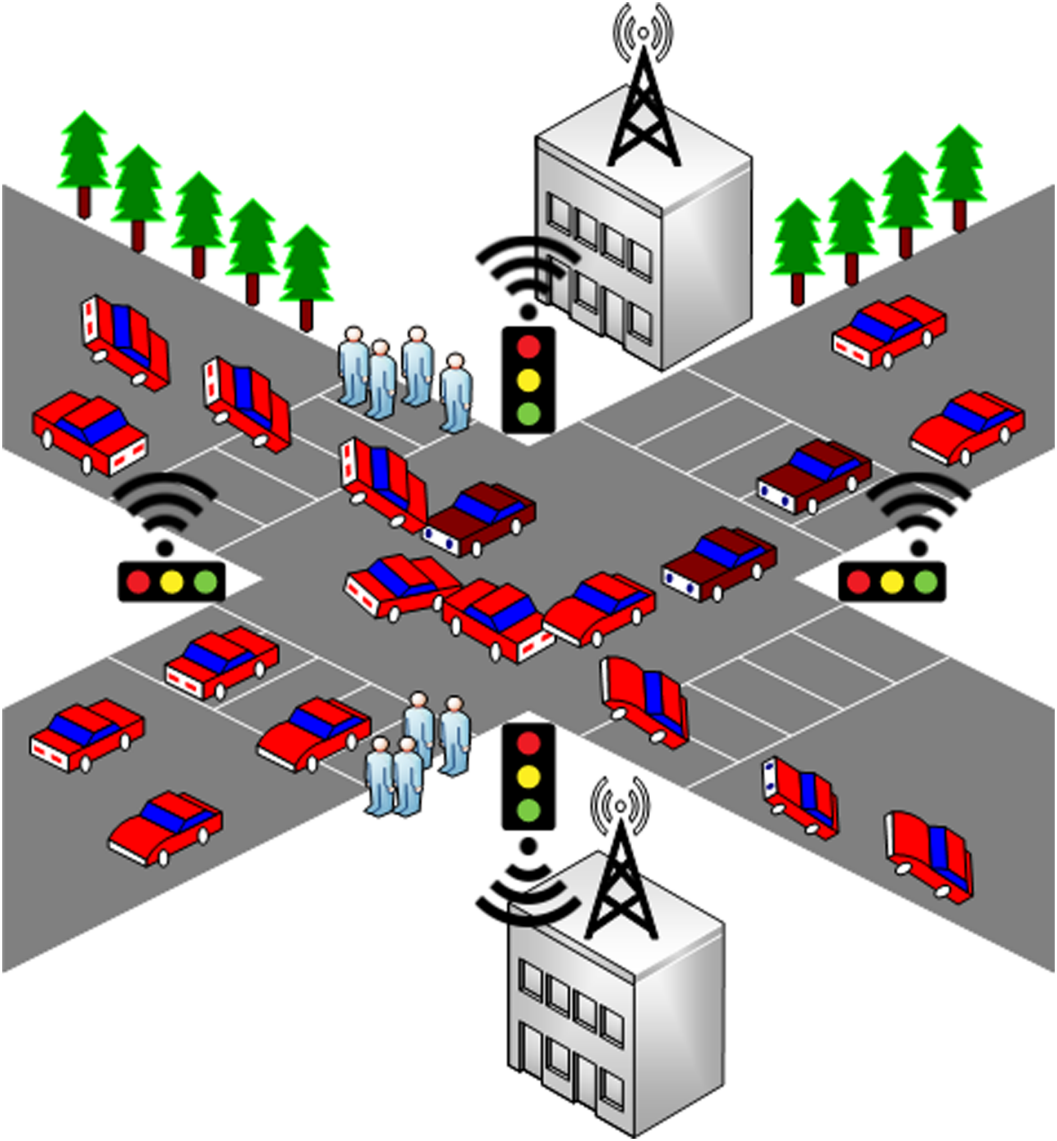
A glimpse of the traffic signal attack and traffic jam.

Figure 3 illustrates that if the security of the TSS compromises, then a big traffic jam can happen in the presence of sensitive vehicles. The users travelling in the red cars and sensitive vehicles are highlighted with maroon color. After attacking the system, cyberpunks successfully created a deadlock so that the government or military officials may be trapped in a traffic jam, especially when they are travelling in the same fleet. In this manner, the criminal elements can easily attack or capture their target. Therefore, this is necessary that the TSS must be secure enough so that the system is not compromised for any reason; particularly during the movement of sensitive automobiles. In the SDAC model, whenever permissions are required to create, action-levels and object-containers are combined for multiple authorization creation. The base of the model is based on the basic entities of RBAC model like object, actions, roles, and users. In this manner, the model security remains tight enough. The assignment of permissions to roles and roles to users is based on attributes so that the model can behave dynamically.

### Formal specification of proposed model

The ALLOY is regarded as an adequate lightweight modeling framework for checking RBAC’s internal accuracy and certain algebraic properties. The conflict-free RBAC was defined using the language ALLOY. The model’s formal specification is as follows.

- ALL_USERS, ALL_ROLES, NET_PERMS, NET_OPRS, and NET_OBJS (all authorized users from every group, all roles, total number of permissions, net number of actions (operations), and net number of objects from every category, respectively).
- USATT, ROATT, PRATT, OPATT, and OBATT denote finite sets of attributes of users, roles, permissions, operations, and objects, respectively.
- OB_CONT and OP_LEV denote object containers for the storage of objects and action level for storing the operations, respectively.
- U_AS ⊆ ALL_USERS × ALL_ROLES: This denotes authorized users to roles assignment with a many to many relationships.
- USER_AS_ROLE: (r : ALL_ROLES) → 2ALL_USERS, the mapping of role r from the set of roles, onto a set of users. This shows roles will be assigned to various users according to the access policy, so that the authorized users can perform their tasks. Besides, the users can only perform those tasks and access those permissions that are assigned to them through roles.
- ASSIGN_USER(r) = {u ∈ ALL_USERS | (u,r) ∈ U_AS}
- P_AS ⊆ NET_PERMS × ALL_ROLES: This shows many-to-many relationships of permission to roles assignment.
- ASSIGN_PERMISSION (r : ALL_ROLES) → 2NET_PERMS, mapping of role r from the set of roles, onto a set of permissions. This shows that permissions are assigned to different roles. In this way, different roles will be designed and assigned to users for fulfilling the organizational tasks.
- ASSIGN_PERMISSION(r) = {p ∈ NET_PERMS | (p, r) ∈ P_AS}
- NET_PERMS = 2(OB_CONT × OP_LEV), this denotes the set of net permissions.
- AUTO_AS_PERMS (atri_r : ALL_ROLES) → 2NET_PERMS, it shows the automatic mapping and assignment of attributed-permissions onto attributed-role atri_r by using attributes. Previously, the administrator used to assign permissions to roles and roles to users, but this model automatically assigns permissions to roles and roles to users by using various attributes.
- AUTO_AS_PERMS (atri_r) = {atri_p ∈ NET_PERMS | (atri_p, atri_r) ∈ P_AS}
- AUTO_AS_ROLES (atri_u : ALL_USERS) → 2ALL_ROLES, it denotes the automatic mapping and assignment of attributed users onto a set of attributed roles atri_r by using attributes.
- AUTO_AS_ROLE (atri_u) = {atri_r ∈ ALL_ROLES | (atri_r, atri_u) ∈ P_AS}
- OP (OP_LV1:OP_LEV) → {OP ⊆ NET_OPRS}, it denotes the mapping of operations onto action-level OP_LV1.
- OB (OB_CT1:OB_CONT) → {OB ⊆ NET_OBJS}, it denotes the mapping of objects onto the object-containers OB_CT1.

## Methodology

The most effective tactic to address the earlier outlined glitches of two concepts (RBAC and ABAC) is given in the recommended model. As discussed earlier, ABAC and RBAC have certain insufficiencies; nonetheless, their union may offer a further stout, tranquil to execute and to manage model. As such, the recommended concept is shared into two parts. From the first part of the model, the involuntary permissions are generated, while in the second portion, the roles are apportioned to users routinely. The permissions are allocated to roles; then, the roles are allotted to users with regards to the requirements. The thorough articulation of the recommended concept with the aid of example scenarios is outlined below in the subsection. Hence, the work is clearly elaborated with the scenario-based case study.

The purpose of the making of diverse action-levels is usually to provide well-ordered access to the objects. In a situation where an object is generated, it is advisable to allot specific quantified actions on that object, for instance, the write and read-only. Particular objects are common in their setting; for that reason, any person could access that object. The administrator could use a given container on the action level, whereas considering the level of contact on those objects. It is evident that an object is generated with the allotment of object attributes and features like IP address, time, and age. In this case, the features of the objects are apportioned to permissions routinely and go-ahead would encompass similar features like objects. It is clear that if an object with the time features similar the object would just be obtainable between 11:00 AM to 3:00 PM. Then, the freshly produced authorization would execute similar function. In addition, when administrator has created the actions with the allotment of attributes like location, username, and designation, so the newly created permissions are also capable of offering these attributes since permission is a combination of object and action. This way, the created permissions are more restricted as compared to the previously proposed models.

**Figure 4:**
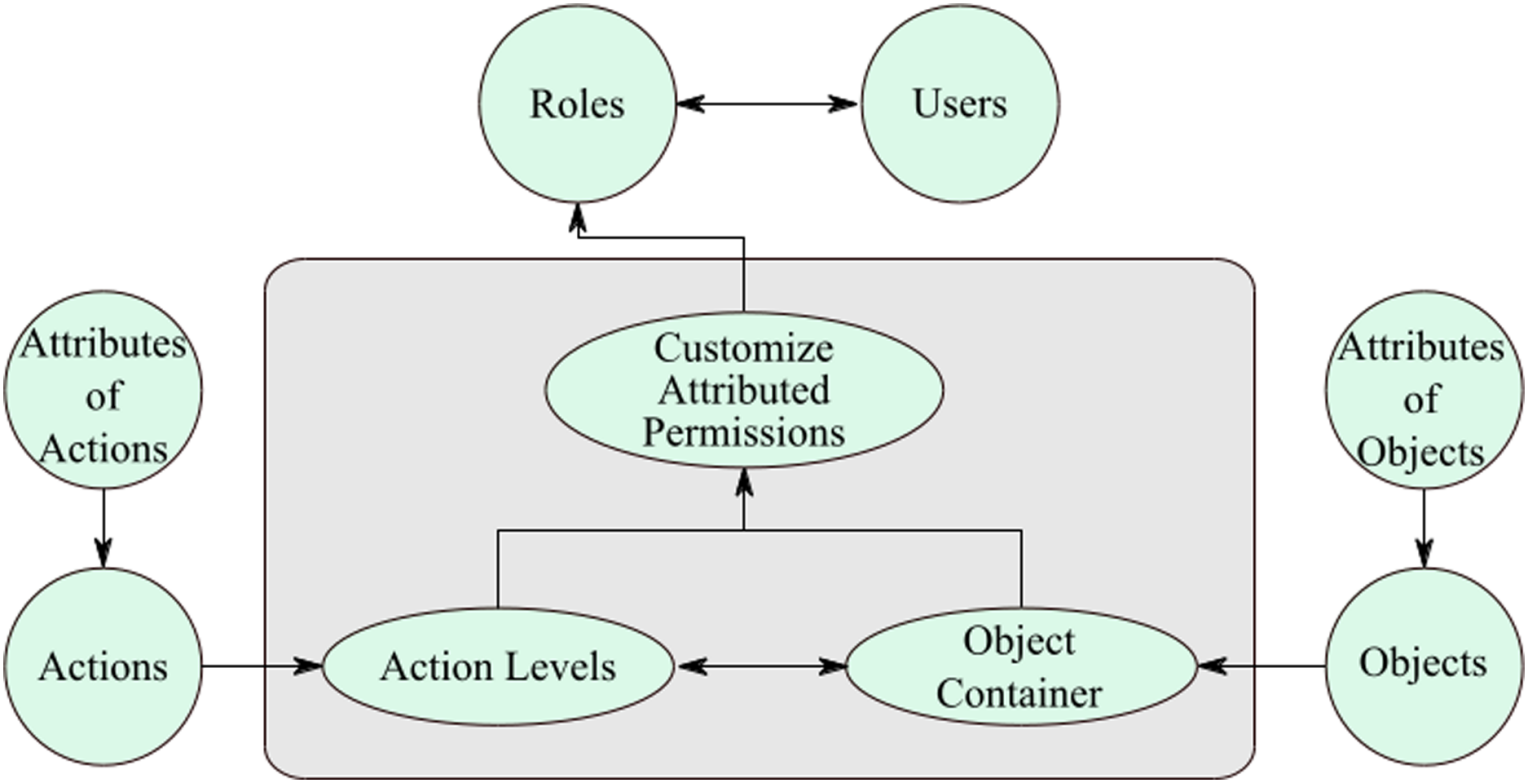
Customize attributed permissions.

It is apparent that a user who has initiated this permission, that user could only execute a specific action on that attributed-object between the apportioned time frame. Moreover, the actions are also attributed to a particular action that can be performed under the given attributes. The authorizations are lively, and any variation in the object and action features would influence the permission’s characteristics. In this case, manual updating is not needed for this authorization creation as the addition of attributes makes the model and permission process dynamic. It is apparent that the model is vibrant giving the ease of authorization generation due to the addition of the attribute on top of stout, and comforts of administration for permission generation attributable to the RBAC model, as shown in Fig. 4.

The permissions are stricter as the attributes of objects and actions are transformed into the permissions. Thus, the customized permissions enforce security in an efficient manner. Such permissions are suitable for sensitive departments like government and military organizations. The organizations that deal with secret information hence the access control should be implemented in a strict way for restricting unauthorized access. Furthermore, the users are restricted to do the task under the given circumstances. According to the recommended model, the customize-permissions are apportioned to roles usually by employing object attributes. For the RBAC model (ANSI/INCITS, 2004), authorizations are allotted manually by the overseer, which is a frenetic work for him/her.

According to the SDAC model, permission apportionment standards are involuntary that would lessen the efforts of overseer or administrator. After the instinctive formation of permissions, the permissions apportioned to roles besides the decision of consignment of permissions is undertaken with the aid of attributes. The object-attributes demarcated on the object creation level under this SDAC model. It is after postulating the object attributes on the object creation stage, and objects are apportioned to object-containers, which is employed on the action-levels. Every security level comprises several attributed-actions, as discussed earlier in this paper. The administrator creates several customized-permissions by applying object-containers and action-levels, which automatically generates permissions. In addition, object and action attributes are copied in the permissions that make the customize-permissions into attributed-permissions.

**Figure 5:**
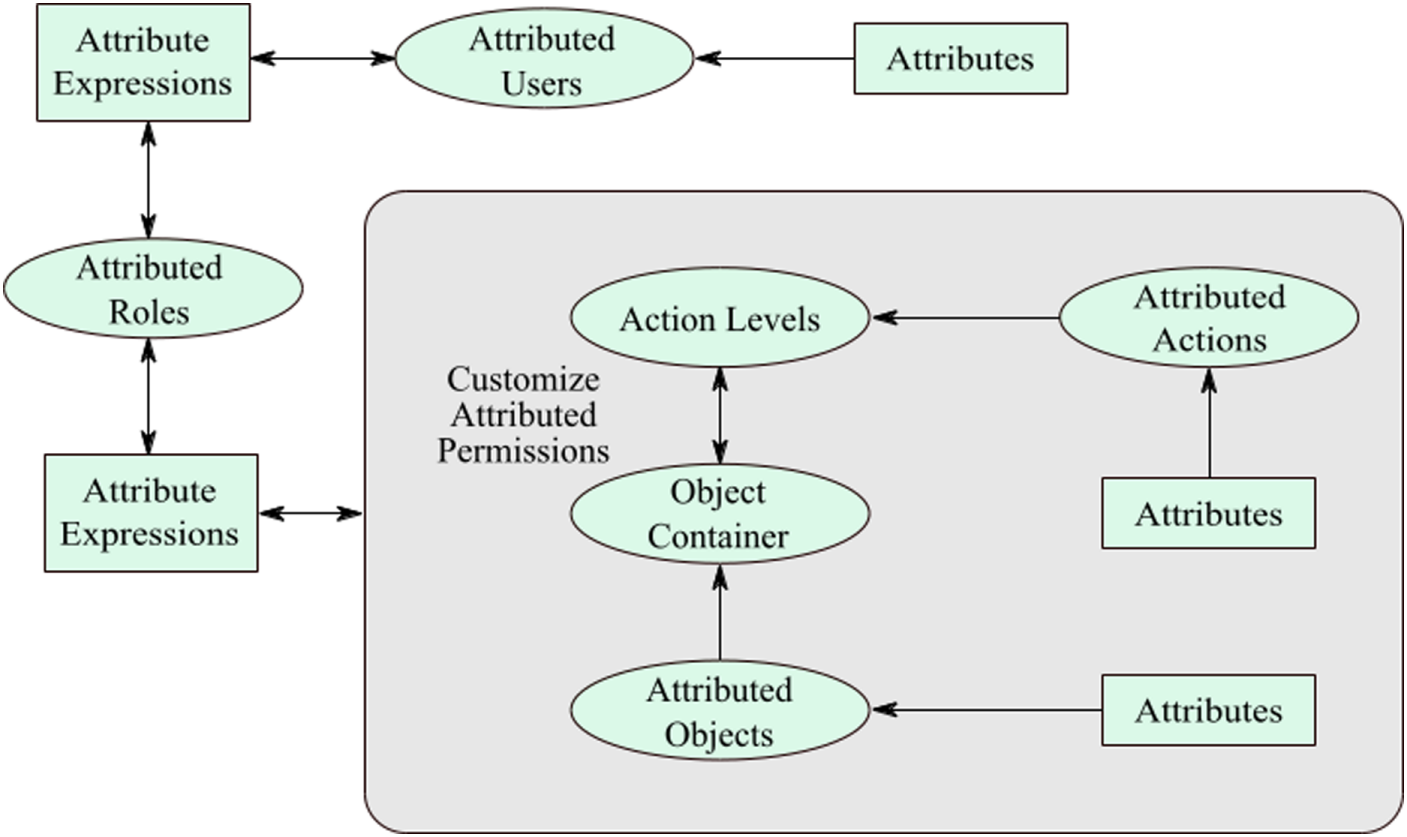
Secure and dynamic access control.

Figure 5 illustrates the complete working of the SDAC model. Here, an administrator creates objects, actions, roles, users, object-containers, and action-levels. While creating the fundamental entities of SDAC model, administrator also assigns attributes to the entities that make them attributed-objects, attributed-actions, attributed-roles, and attributed-users. In the next step, administrator assigns the attributed-objects to object-containers and attributed-actions to action-levels, so that administrator can create multiple customize attributed permissions. As the permissions, roles, and users are attributed, the system starts matching the attributes. The system automatically assigns the permissions to roles and roles to users by matching their attributes. In addition, the delegation of rights to users and user revocation process is dynamic, since the system grants or revokes access for a user by matching the attributes of users. For example, if a user has attributes username = ‘Bob’, IP address = 192.168.1.21, and time = 9:00 am to 6:00 pm, then the user can access the resources with the same attributes as well as attribute values. If the user wants to access after 6:00 pm, then users receive the message of access is denied that means users rights are revoked. So, if a user is part of the organization then the user must fulfill the access policy and attributes values; otherwise, the user cannot be able to access or perform its tasks. In short, if user’s attributes change then rights also change. In this manner, administrator workload is reduced as this part is dynamically performed for assignment of permissions and roles.

**Figure 6:**
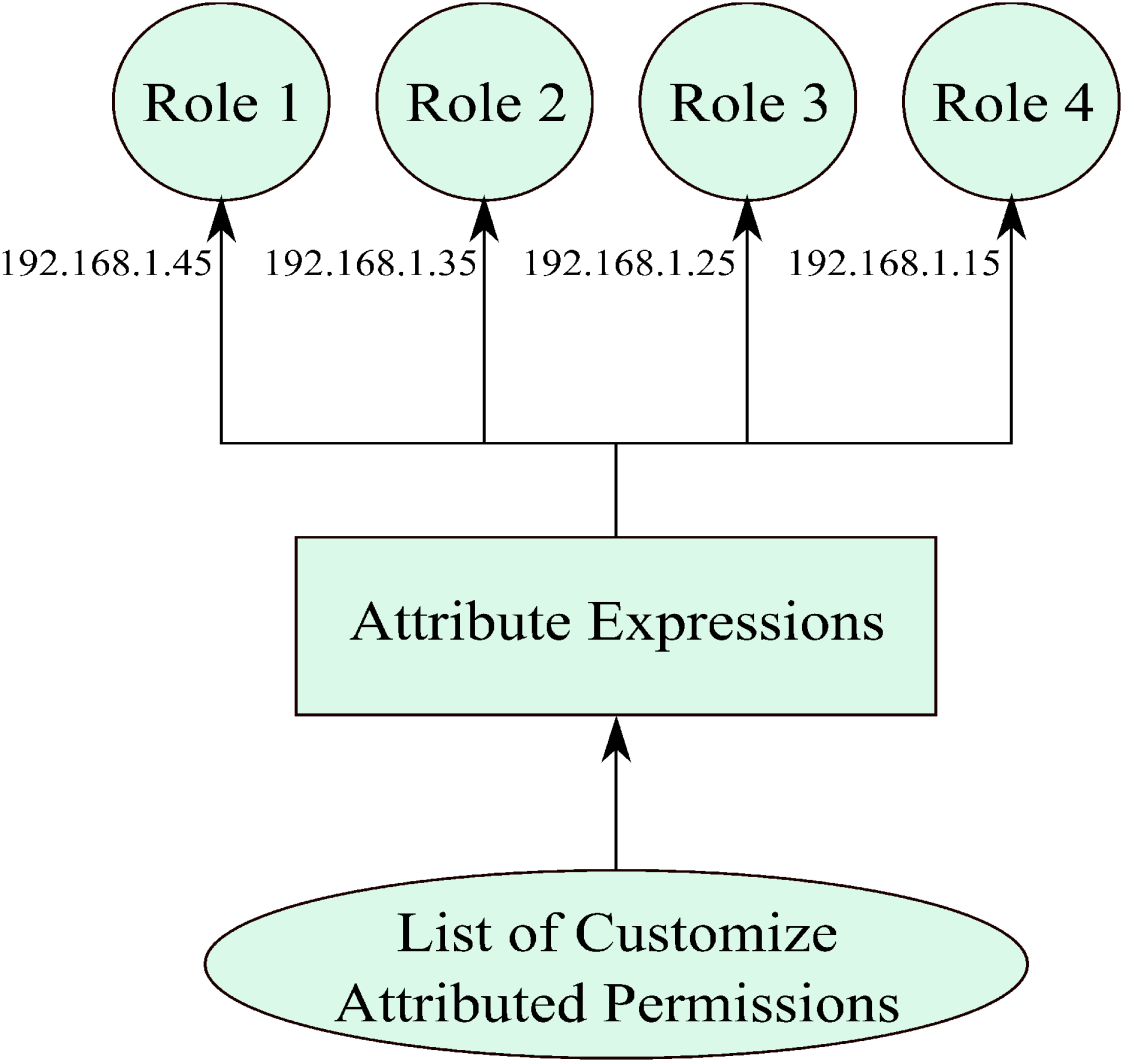
A view of attributed-permissions to attributed-roles assignment.

**Figure 7:**
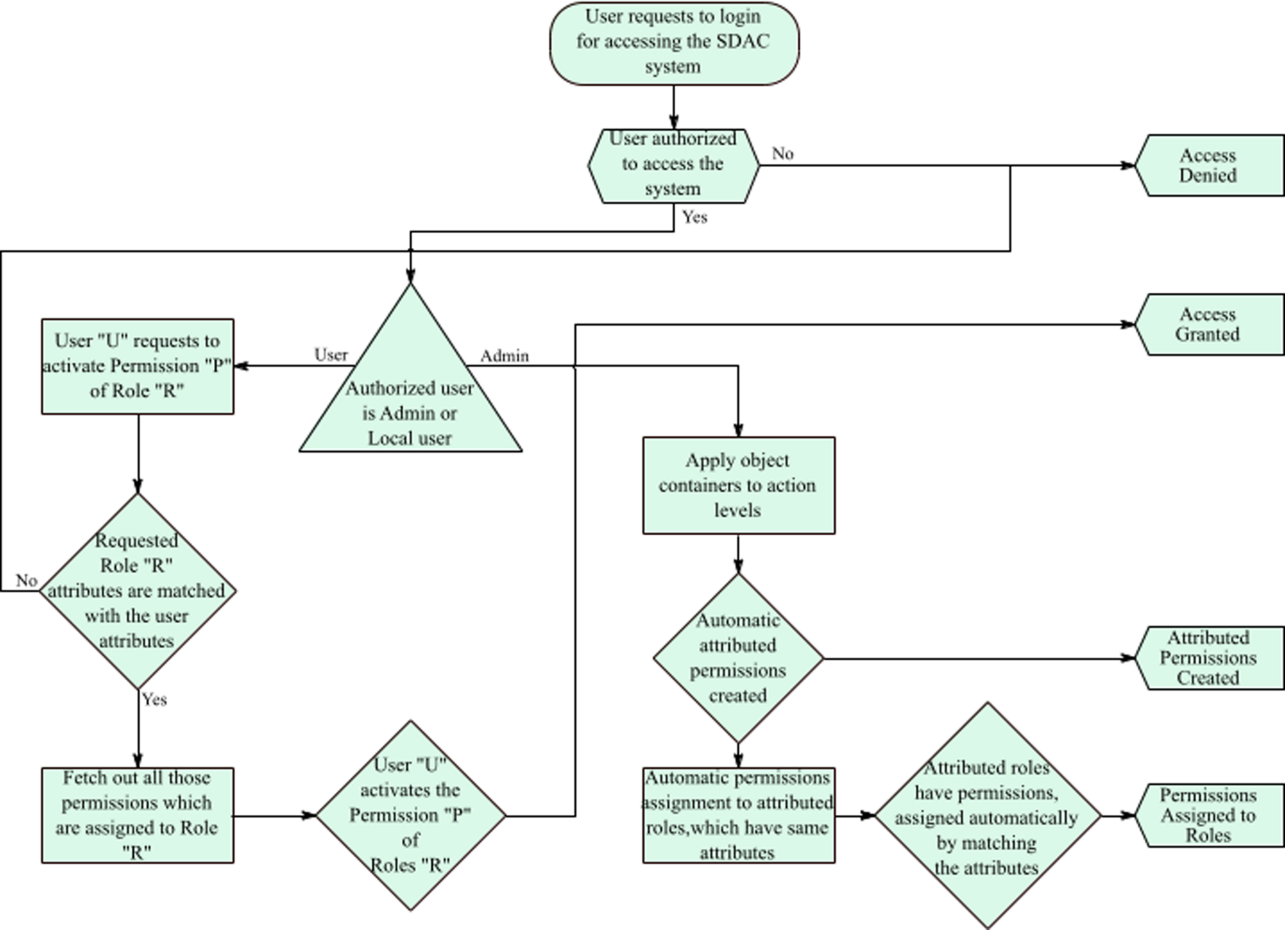
Flow chart of the SDAC model.

The dynamic behavior of this work is due to the addition of the attributes, but previously administrator used to do this manually. As the permissions are automatically formed, individual permissions are to be allocated to describe roles as presented in Fig. 6. Endorsement of transmissions to parts is done by permissions attributes. The attributed-roles are based on the period of the role construction; the administrator describes attributes of a role. A consignment of consents to roles is, therefore, performed by pairing these traits. For instance, a task that was fashioned with an attribute IP address has the value of 192.168.1.35, meaning that all individuals or groups with permission of the same attribute value automatically are assigned this particular role, as shown in Fig. 6. Similarly, system matches the permissions with other roles and assigns the permissions to roles that have the same IP-address attribute. Lastly, the flowchart of this work is also attached for a broader view of the model (Fig. 7).

In the last, the algorithm of the proposed model is described in five steps.

**Step1:** Basic entities creation for SDAC model: attributed-objects, attributed-actions, attributed-roles, and attributed-users

**Algorithm 1:** Create attributed-objects (attributed-roles or attributed-actions) entities.

1: **input** <- the object name (role name or action name) with attributes
2: **validate** the user input on object name (role name or action name) field and ip address field
3: **add** the object (role or action) with fields as user input
4: **update** the object (role or action) to the database

**Algorithm 2:** User creation and user login process.

1: **input** <- the username, password with attributes
2: **validate** the user input on object name (role name or action name) field and ip address field
3: **if** users are in login page then
4: **check** the user is logging to admin type
5: **verify** the username and the password from user input
6: **check** if users’ information matches with the information created before (period time, IP address)
7: **if** all information is matched, load the admin/user page
8: **end if**
9: **if** users are in register page then
10: **add** the user with fields as user input
11: **update** the user to the database
12: **end if**

**Step2:** Creation of object-containers and attributed-objects assignment to these containers. Creation of action-levels and assignment of attributed-actions to different action levels.

**Step3:** Multiple customize-permissions creations by applying action-levels on object-containers

**Algorithm 3:** Multiple permissions creation.

1: **load** all available object-containers and action-levels to comboboxes
2: **initialize** an integer count variable with a value of 0
3: **for** action in selected action-level do
4: **for** object in selected object-container do
5: **add** the permission with the action and the object
6: **if** this permission is existed then
7: **continue**
8: **else**
9: **update** the permission with the related action and object to the database
10: **increase** count by 1
11: **end if**
12: **end for**
13: **end for**
14: **return** count (as the number of permissions that are created)

**Step4:** Customize attributed-permissions automatically assigned to attributed-roles and attributed-roles are assigned to attributed-users with the help of attributes.

**Algorithm 4:** Customize-attributed-permissions automatically assigned to user with the help of attributes.

1: **load** all available permissions to the array list “permiss”
2: **for** permission in permiss do
3: **check** if the login time is matched with the required period time **for** the object in permission
4: **check** if the user’s Ip Address matched with required period time for the object in permission
5: **if** both conditions are satisfied then
6: **add** the permission to the array list “privilegedPermissions”
7: **end if**
8: **end for**

**Algorithm 5:** Attributed-roles automatically assigned to attributed-user with the help of attributes.

1: **load** all attributed-roles to the array list “roles”
2: **for** role in roles do
3: **check** if the login time is matched with the required period time for the role
4: **check** if the user’s IP Address matched with required period time for the role
5: **if** both conditions are satisfied then
6: **add** the role to the array list “privilegedRoles”
7: **end if**
8: **end for**

**Step5:** Users can access the assigned attributed-roles and permissions against every role.

### Example scenarios

In this subsection, we have elaborated on two different example-based scenarios so that the concept of this work can be easily provided.

### Learning management system

The first case study has been taken from the scenario of an education department using a learning management system (LMS). Through the use of LMS, teachers are privileged with giving online quizzes, assignments, providing study materials, and taking attendance. On the other hand, students depend upon teachers and teachers to control the activities and authority of students in the LMS. In conclusion, LMS distinguishes teachers’ and students’ rights as different. For example, providing the authority to teachers so that they can create different educational documents for students; such as slides, pdf, word document, and other study materials. On the condition that a teacher wants to assign a file (like a.pdf) to all students, the teacher is granted the right to access all groups of students or a single student. Additionally, the teacher gets the advantages of defining the authority of each level of student to the file or files uploaded. This shows that the two roles are involved in this action, and the roles are student and teacher.

A teacher can upload files such as slides, quizzes, and results. Moreover, the teacher can also modify files like editing quizzes, deleting the assignment, or replacing files. On the other hand, student’s permissions are: to download the study material, upload assignments, solve online quizzes, and check their marks from anyplace. The students and teachers can only access the resources only after logging into the system so that their identity and authority level can be verified. This way, the educational files are uploaded with the attribute ‘username’, for students to easily access the data from inside and outside of college or university. In the meantime, of attendance and quizzes, files are composed of the attributes ‘IP-address’, ‘date’, and ‘time’. This allows a student to mark attendance only when in class, on a particular date and time.

The availability of exam files is only specific to scholars that are verified in the college or university system, which can all be confirmed from an IP range. Now, consider the situation from the initial point of the SDAC model (objects and actions creation). The teachers add PowerPoint slides, course files, books.pdf, and they are asked to assign attributes value over the files. As the educational-materials are bound for all students, therefore it can be opened, edited, read, and downloaded from the scholars’ side. These records are further added to different object-containers. On the other hand, different actions are facilitated with various attributes. Then actions are assigned to different action-levels. Now, the action-levels are applied to the object-containers for the automatic formation of customized permissions. The example of objects with different attributes, object-containers, action with different attributes, and action-levels is given in Table 1, for better understanding.

**Table 1:** Object containers and action levels.

Object name	Attribute	Object container	Action name	Attribute	Action level
File1.txt	Location	Container1	Read, Write, Edit	Time & Username	Level1
File2.exe	Location	Container2	Read, Download, Delete	Username & Date	Level2
File3.ppt	Location	Container3	Delete, Write, Submit	Location & Designation	Level3
File4.doc	Date & Time	Container3	Delete, Write, Submit	Location & Designation	Level3
File5.xlsx	Date & Time	Container3	Delete, Write, Submit	Location & Designation	Level3

When Container1 is combined with Level1 then three different permissions are formed. The attributes of objects and actions are also transferred in the newly created permissions. In the next step, Container2 is employed to Level2 which creates three attributed-permissions at once. After this creation, Container3 is applied on Level3 that creates nine attributed-permissions in the result. Container 3 has three attributed-objects and Level3 also has three attributed-actions. In this way, nine (3 × 3 = 9) permissions are created. Container1 consists of one object ‘File1.txt’ and the users can access this object from the given locations only. Moreover, users can perform actions read, write and edit on this object only within the given time domain and authorized usernames. In this way, these permissions are stricter as compared to the previous model permissions. On the other hand, Container2 object is also accessible from the verified location attribute as well as the actions read, download, and delete can only be performed when the attributes username and date are fulfilled.

In the final permission creation process, there are three different objects in Container3. File3.ppt can be accessible from the given location attribute. File4.doc and File5.xlsx are accessible with the given time and date. In addition, the users can perform actions delete, write, and submit on Container 3's objects, but the location and designation of the users should be correct. As the permissions are attributed and roles are also facilitated with different attributes so the permissions are assigned to roles by matching their attributes. An example is given in Table 2 for a clear understanding. Permissions prms1 to prms9 are allocated dynamically to Role1 as they have attribute, i.e., location. Permissions prms10 to prms15, therefore, get to be allocated to Role2, as they have the same attribute and attribute value, i.e., date and time. Likewise, system allocates Role1 to users’ User1 to User7’, as their attribute values are matched. In addition, Role2 is assigned to users’ User8 to User15’ with the help of their attributes.

**Table 2:** Permissions assignment to roles and roles to users.

Permission name	Permission description	Permissions attributes	Role name	Role attributes	Users	Attributes of users
Prms1	Read, File1.txt	Location	Role1	Location	User1,…User7	Location
Prms2	Write, File1.txt	Location	Role1	Location	User1,…User7	Location
Prms3	Edit, File1.txt	Location	Role1	Location	User1,…User7	Location
Prms4	Read, File2.exe	Location	Role1	Location	User1,…User7	Location
Prms5	Download, File2.exe	Location	Role1	Location	User1,…User7	Location
Prms6	Delete, File2.exe	Location	Role1	Location	User1,…User7	Location
Prms7	Delete, File3.ppt	Location	Role1	Location	User1,…User7	Location
Prms8	Write, File3.ppt	Location	Role1	Location	User1,…User7	Location
Prms9	Submit, File3.ppt	Location	Role1	Location	User1,…User7	Location
Prms10	Delete, File4.doc	Date & Time	Role2	Date & Time	User8,…User15	Date & Time
Prms11	Write, File4.doc	Date & Time	Role2	Date & Time	User8,…User15	Date & Time
Prms12	Submit, File4.doc	Date & Time	Role2	Date & Time	User8,…User15	Date & Time
Prms13	Delete, File5.xlsx	Date & Time	Role2	Date & Time	User8,…User15	Date & Time
Prms14	Write, File5.xlsx	Date & Time	Role2	Date & Time	User8,…User15	Date & Time
Prms15	Submit, File5.xlsx	Date & Time	Role2	Date & Time	User8,…User15	Date & Time

### Traffic control system

The second case study has been taken from the scenario of a traffic control system (TCS) using IoT-based traffic signals. Through the use of TCS, users are privileged with setting the timing for various signals, block some roads for security/emergency reasons, setting green lights for a complete route for military/government officials, and monitoring TCS. On the other hand, administrator can set the access rights for various users for controlling the activities. Resultantly, TCS distinguishes administrators and end-users rights as different. The end-users need so that they can control and deal with different activities for TCS; such as, set Timers, block a route, open a route, and other related activities. For this purpose, the administrator assigns various access rights to the users, like some users, can set timers for various routes, some users can block/open some routes for the safety measures of sensitive vehicles, and some users can approve the timing as well as blocking/opening of routes.

The administrator will set different rights for different users according to their ranks and security levels. Only the authorized users can access the system that means the users will access the system after providing their credentials. The administrator assigns the authority to users User1 to User6 for setting the timers for TCS, User7 to User10 for blocking/opening the routes, and User11 to User15, to approve/monitor all the tasks. In this way, the user tasks, as well as the approval and monitoring tasks dealing with other users, will tighten the security. In addition, the administrator can control the violation of segregation problems up to some extent. A complete view of the objects, actions, permissions, roles, users, and their particular attributes provided in Table 3. Here, the understanding of the users' access rights with detail can be achieved.

**Table 3:** Another view of permissions assignment to roles and roles to users in TCS.

Permission name	Action, object	Permissions attributes	Role name	Role attributes	Users	Attributes of users
Prms1	View, Timers.txt	Location	Role1	Location	User1,…User6	Location
Prms2	Write, Timer.txt	Location	Role1	Location	User1,…User6	Location
Prms3	Edit, Timer.txt	Location	Role1	Location	User1,…User6	Location
Prms4	Submit, Timers.txt	Location	Role1	Location	User1,…User6	Location
Prms5	View, Blockroute.txt	Username	Role2	Username	User7,…User10	Username
Prms6	Write, Blockroute.txt	Username	Role2	Username	User7,…User10	Username
Prms7	Edit, Blockroute.txt	Username	Role2	Username	User7,…User10	Username
Prms8	Submit, Blockroute.txt	Username	Role2	Username	User7,…User10	Username
Prms9	View, Openroute.txt	Username	Role2	Username	User7,…User10	Username
Prms10	Write, Openroute.txt	Username	Role2	Username	User7,…User10	Username
Prms11	Edit, Openroute.txt	Username	Role2	Username	User7,…User10	Username
Prms12	Submit, Openroute.txt	Username	Role2	Username	User7,…User10	Username
Prms13	Approve, Blockroute.txt	Designation	Role3	Designation	User11,…User15	Designation
Prms13	Deny, Blockroute.txt	Designation	Role3	Designation	User11,…User15	Designation
Prms14	Approve, Openroute.txt	Designation	Role3	Designation	User11,…User15	Designation
Prms15	Deny, Openroute.txt	Designation	Role3	Designation	User11,…User15	Designation

### Benefits

SDAC model inherits the features of basic RBAC entities like objects, actions, permissions, roles, and users. Due to these entities, this model is secure and it implements the concept of least privilege (LP) in the SDAC model. Usually, the RBAC model facilitates the organization with LP, but the proposed model is more suitable for LP as it further decreases the access rights of the users. For instance, if a user wants to obtain access to some specified resources then, in RBAC system, all the necessary permissions are assigned to him/her permanently, or we can say that the user can access those permissions all the time. On the other hand, SDAC model is implemented with attributes like date, IP address, time, and username, etc. Therefore, a user can only access the permissions by fulfilling the implemented attributes. For example, if a user has permissions on a resource and the date attribute is set for these granted permissions. The user can only access the permissions on the assigned dates; however, in a typical RBAC model, the user can access the permissions all the time. Therefore, for security concerns, the proposed model serves effectively than the typical RBAC and ABAC models.

The SDAC model is dynamic because of the addition of attributes. Commonly, in the RBAC model, all the tasks are performed manually by the administrator. If any change is needed, the administrator is troubled again since administrator has to work over it once more. This model decreases the burden of the administrator because after creating the objects and actions, the administrator can create number of permissions with a single click. The attributed permissions are automatically assigned to roles and roles are assigned to users on the basis of roles and user attributes. Dealing with the changes in the access control system is another vital issue to be discussed. If the administrator wants to change the access rights of users towards roles, the administrator simply has to change the attributes of the roles. All the users automatically achieve access to the roles according to the new attributes values or unique attributes–reducing the workload of administrator.

### Limitations

Even though the proposed model is comparatively effective than the typical RBAC or ABAC models, there are some limitations that can be considered. Firstly, SDAC model is facilitating the permissions to roles assignment on the basis of attributes. This model is not covering the conflicted permissions and conflicted roles feature. This is a limitation regarding conflict of interest because a user can hold both conflicted permissions and there is no restriction for this necessary parameter of access control. The second limitation is that the administrator has to spend more time on the object and action creation stage, as compared to the typical RBAC model. Thirdly, the permissions are assigned to roles on the basis of various attributes. However, permissions are assigned permanently.

In the ABAC system, the whole model is dynamic, so this model is not facilitating the change on the permissions’ end, as if administrator wants to delete permissions from a role or assign them to another role then administrator has to work from the start. The administrator deletes the permissions manually and again generates the permissions with the new attributes so that the newly generated permissions can be assigned to new roles. Such limitations in the proposed model are implied because model must respond to all kinds of changes on the basis of attributes. But, this model is not facilitating the permissions to roles end. However, the authors aim to address these limitations in future work.

## Simulation and discussion

**Figure 8:**
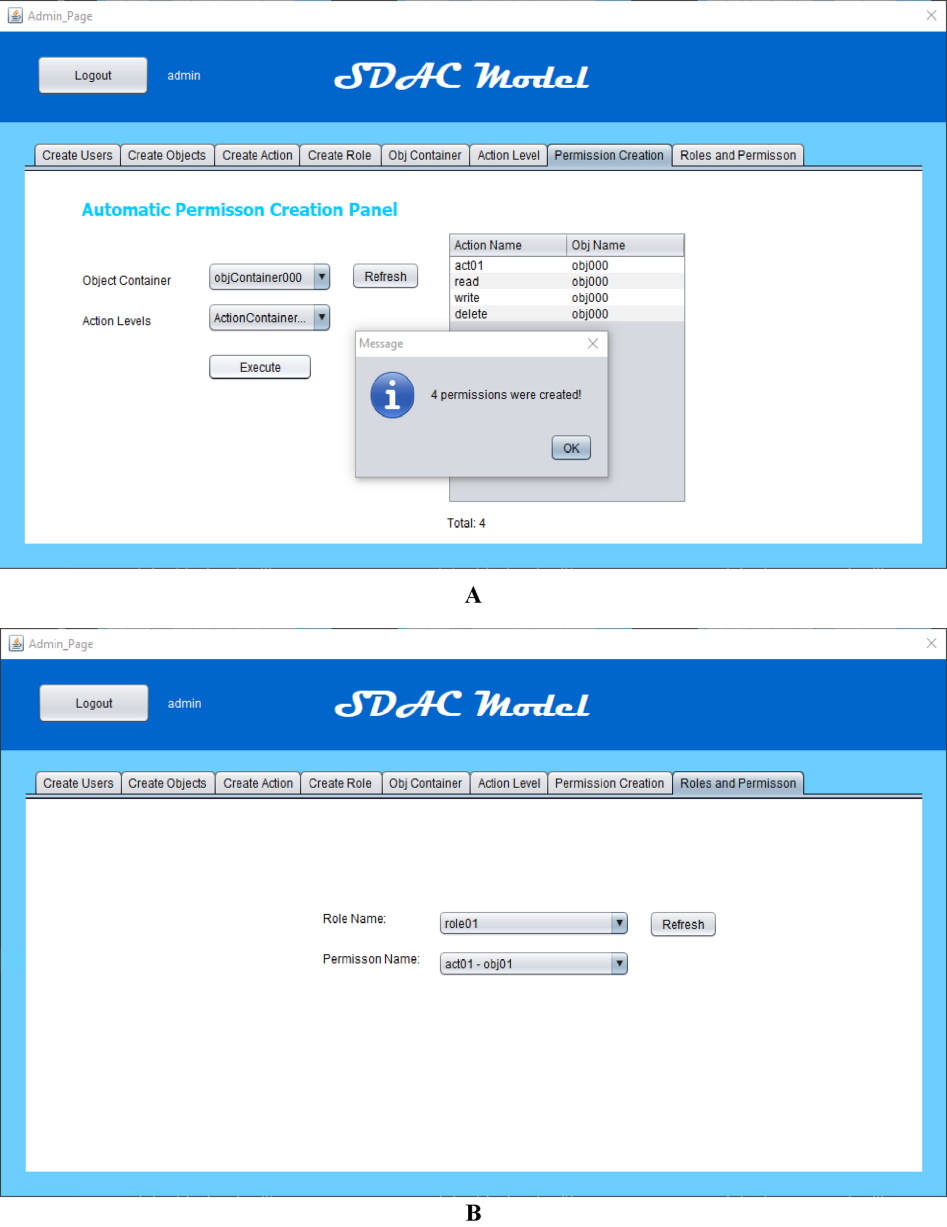
(A) Multiple permission creation (B) roles and permission view.

In this section, we have presented the simulation-based study of the SDAC model. Here, the working, performance, and the concept of this work are clearly demonstrated. For the sake of simplicity and clarity, we have shown the simulation and results with discussion. When object containers and action levels are created, then the admin can create a number of permissions automatically. The automatic permission creation is performed through the execution of object containers over action levels. If an object container contains five objects and the targeted action level contains seven actions, this execution creates 35 permissions. When admin presses the execution button after selecting the object container and action level, then the permission list displays in grid view after creation. The screenshot of permission creation panel is attached in Fig. 8A.

Furthermore, the administrator can also create roles with specifying role attributes like days, time, IP address, and designation, etc. As role is the bridge between permissions and users, and we use this bridge for the automatic assignment of permissions to roles and automatic role assignment to users on the basis of attributes or by matching their attributes. A permission is a combination of object and action. Every object has attributes in this application. After the permission creation, object attributes of every permission match with roles attributes, and all the permissions which have same values of object and role attributes are assigned to those roles. The role creation panel and roles view with their assigned permissions are given in Fig. 8B. On the other hand, the end-users can access the roles as well as assigned permissions after logging into the system. In this way, only authorized users can access the system by giving their username and password for authentication. Then the system will show only those roles and permissions, whose attributes are matched with users’ attributes.

The software is being quickly tested on Windows 10 OS Version 1903 (build 18362.719). The source code of the simulated work is in Java language. The application is built using the NetBeans IDE 8.2 (Build 201609300101). JDK 1.8.0_111 is the variant of the framework construct programming environment. The UserForm class is the primary class in this project. In this context, the program with database link is checked using Microsoft SQL Server 2017, with information about the framework version as follows:

### Software/environment

- Operating System: Windows 10 64 bit Version 1903 (build 18362.719)
- IDE: Netbeans IDE 8.2
- Java Development Kit: 1.8.0_111
- Database Management System: Microsoft SQL Server 2017 (RTM) - 14.0.1000.169 (X64)
- Java Database Connectivity Driver: Microsoft JDBC Driver 6.2 for SQL Server

### Hardware

- Processor: Intel® Core i5 9300H
- Memory: 12288 MB of RAM
- Hardisk: Samsung 256 GB PCI EXPRESS Solid State Drives
- Chipset: Intel® 300 Series

**Figure 9:**
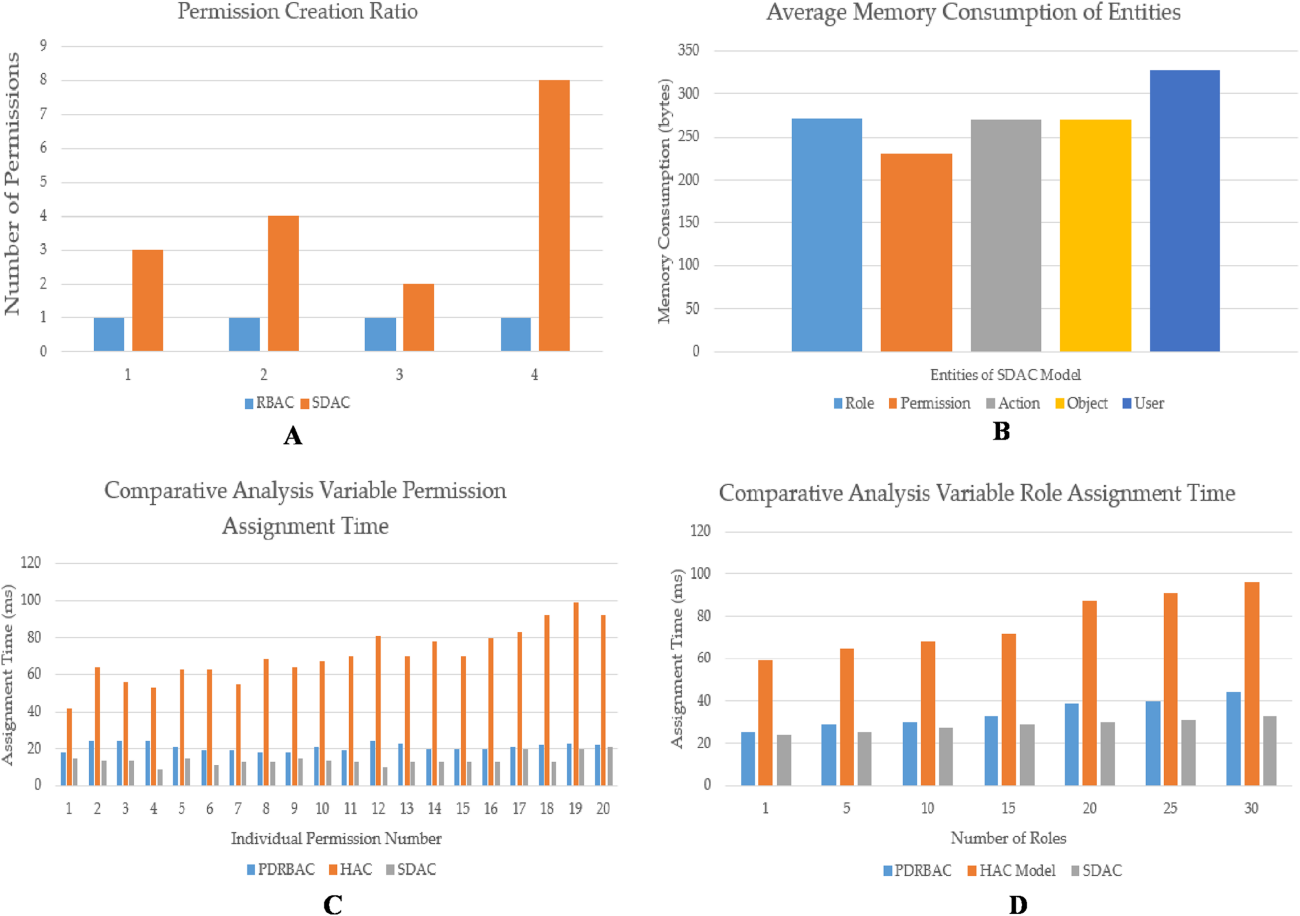
Analysis of SDAC model with respect to various aspects. (A) Administrator efforts for permission creation. (B) Average memory consumption of each entity. (C) Variable permission assignment time. (D) Variable role assignment time.

The SDAC model for the IoT-based TSS allows the administrators or authorities to efficiently implement access control by creating multiple permissions at the same time. On the contrary, typical RBAC will enable administrators to create permissions one by one. The results are presented in Fig. 9A and compared with the RBAC model. In Fig. 9A, it is observed that the SDAC model is creating more permissions in every attempt of permission creation. The administrator successfully created multiple permissions in SDAC model (in orange colour bars). In the first attempt, three permissions are created with the merger of object containers and action levels (3 objects × 1 action = 3 permissions). In addition, the second attempt has four permissions that are created by applying object container (2 objects) with action level (2 actions). Furthermore, two permissions are created by applying object container (2 objects) with action level (1 action). Moreover, eight permissions are created by merging object container (4 objects) with action level (2 actions). In comparison, the administrator has created permission one by one in a typical RBAC model (in blue colour bars). In this manner, the administrator can create more permissions through this model as compared to the standard RBAC model.

The results are compiled on the basis of the simulated application and proposed model working criteria. The reason behind more permission creation in our more model is the applying of the object containers with various action levels. In this way, the administrator may build several permissions at the same time. The screenshots of the developed system are also shown in Figs. 8A and 8B. On the other hand, the analysis of the proposed SDAC model has also improved performance concerning memory consumption, role assignment, and permission assignment time. The memory consumption of each entity is provided in Fig. 9B.

Each entity is highlighted with different colors such as role (sky blue bar), permission (brown bar), action (grey bar), object (yellow bar), and user memory consumption (dark blue bar). In addition, the variable processing time is calculated against each permission assignment time and given in Fig. 9C. Moreover, the permission assignment time is compared with other similar models such as permission-based dynamic RBAC (PDRBAC) (Aftab et al., 2019) and hybrid access control model (HAC) (Aftab et al., 2020). The variable permission assignment time of PDRBAC (blue bars) is comparatively better than the HAC model (brown bars). On the other hand, our proposed SDAC model (grey bars) performed significantly better as compared to previously proposed models. Furthermore, the variable role assignment time is also calculated against more than one role. The number of roles and their assignment time can be viewed from Fig. 9D. The role assignment time of PDRBAC (blue bars) is comparatively less than the HAC model (brown bars). On the contrary, SDAC (grey bars) performs well and assigns roles while taking less time, as compared to previous models (PDRBAC and HAC).

## Conclusion and future work

Primarily, this paper purposes a novel approach by joining RBAC and ABAC and relishing the paybacks of two models besides covering their scarcities. SDAC model helps to reduce administrator loads since all the tasks, including permission creation, are more efficient, unlike the traditional RBAC model. Furthermore, the permissions to roles assignment and roles to user assignment are dynamic by utilizing the concept of attributes. The prototypical illustration helps in providing dynamicity and automatic role organizing facilities. The proposed SDAC model is more secure as it is based on entities of the RBAC model. In addition, the permissions of this work are stricter and more complicated due to the attributes of objects and actions. In comparison, the SDAC is dynamic because of the addition of attributes that decreases the administrative burden and makes it easier to use for the system administrator. In the future, our team will consider the prospect of exploring the security of internet of connected vehicles and localization systems.

## Additional Information and Declarations

### Data Availability

The following information was supplied regarding data availability: This code is available at https://github.com/sdacModel/sdacModel.
